# Supplementary material for: Sharing results with participants (and community) in malaria related research: Perspectives and experience from researchers
Source: PLOS Glob Public Health. 2023 Sep 5;3(9):e0002062. doi: 10.1371/journal.pgph.0002062 (PMC10479888; doi:10.1371/journal.pgph.0002062)
Supplement: S2 Appendix — (PDF) [file pgph.0002062.s002.pdf]

Interview Guide- CONSENT

Hi, thanks for being here today. My name is [xxxxx] and I am a clinical researcher from Menzies School of Health Research. Alongside [xxxxxx], we are conducting mixed methods research into the practice of dissemination of results to participants following clinical trials. You have been selected as a specialist in malaria clinical trials.

We would like to interview you to understand your experiences and your opinions surrounding the current practice of dissemination of these results. These expert interviews will provide the basis for a quantitative survey instrument to distribute to the investigators of global clinical trials in malaria over the last 15 years.

Before we continue, I need to ask your consent to participate in this project:

And that you understand the information that was sent to you about the project, agree to take part in this study, understand that you can ask questions at any time, and understand that you are free to withdraw (This means you can say NO) at any time.

If you have any concerns or complaints regarding the ethical conduct of the study, you can contact Human Research Ethics Committee of the NT Department of Health and Menzies School of Health Research.

☐ [NAME], do you agree to participate in a telephone interview about dissemination of clinical trial results?

☐ [NAME] Do you agree to have your voice recorded during the interview and transcribed for analysis?

Interview Guide

To start, can you tell me about how you disseminate your research results after a clinical trial in general?

To what audiences and how?

[PROMPT]: Who do you target when disseminating trial results?

In your most recent clinical trial, how did you disseminate the results?

How did you do this for the trial participants?

What were the main challenges?

What do you think worked well?

Did you use a similar or different approach to what you have used in the past?

[PROMPT]: Are there any reasons why you didn't directly target the participants?

Can you think of some reasons why researchers may not disseminate the results to trial participants?

[PROMPT]: Can you think of other reasons why researchers may not disseminate the results to participants?

Are there any reasons why you didn't directly target the participants? What do you think are reasons not to disseminate?

Can you think of other reasons why researchers may not disseminate the results to participants?

What is your understanding of the ethical implications or requirements for disseminating clinical trial results to participants?

Before commencing a trial, say in the proposal and early stages, is dissemination at the completion of the trial a consideration for you?

Thank you for taking the time to tell me about your research. Is there anything else you would like to share?
