# Supplementary material for: Sharing results with participants (and community) in malaria related research: Perspectives and experience from researchers
Source: PLOS Glob Public Health. 2023 Sep 5;3(9):e0002062. doi: 10.1371/journal.pgph.0002062 (PMC10479888; doi:10.1371/journal.pgph.0002062)
Supplement: S3 Appendix — (PDF) [file pgph.0002062.s003.pdf]

Disseminating Results to Participants

Start of Block: Default Question Block

Q1 Think about the last trial you completed. Who did you report study results to? Select all that apply

1. Ministry of Health (1)
2. Funders (2)
3. Community Leader/s (3)
4. Trial Participants (4)
5. Local Health Authorities (5)
6. Peer reviewed publication/conference (6)
7. Institutional Ethics Review Board (7)
8. Study Clinicians (8)
9. Other Study Staff (9)
10. Other (10) ____

Q2 Do you think results should be communicated regardless of the outcome of the trial?

- Definitely yes (1)
- Probably yes (2)
- Probably not (3)
- Definitely not (4)
- Not sure (5)

Q3 After a clinical trial, how important is it to you to inform participants of the results of the trial?

- Not Important at all (1)
- A little important (2)

- Mostly Important (3)
- Extremely Important (4)

Q4 In your last 3-4 trials, have you targeted trial participants in dissemination of results?

- Yes (1)
- No (2)
- In some trials, but not all (3)
- Not sure (4)

Display This Question:

If In your last 3-4 trials, have you targeted trial participants in dissemination of results? = No

Or In your last 3-4 trials, have you targeted trial participants in dissemination of results? = In some trials, but not all

Q5 What is the MAIN reason why not?

- It is not a priority for me (1)
- Financial restraints (2)
- Time between analysis and completion of study was too long (3)
- Lack of interest from my institution (4)
- I don't think the community is interested (5)
- Other (6) ____

Display This Question:

If In your last 3-4 trials, have you targeted trial participants in dissemination of results? = Yes

Or In your last 3-4 trials, have you targeted trial participants in dissemination of results? = In some trials, but not all

Q7 What methods did you use to disseminate your results to participants? Select all that apply

11. Role Play (1)
12. General Community Meeting (2)
13. Participants Only Meeting (3)
14. Individual Phone Calls (4)
15. Generic Email to Participants (5)
16. Social Media Campaign (6)
17. Other (7) ____

Display This Question:

If In your last 3-4 trials, have you targeted trial participants in dissemination of results? = Yes

Or In your last 3-4 trials, have you targeted trial participants in dissemination of results? = In some trials, but not all

Q8 What are some challenges you have faced in coordinating dissemination to participants? Select all that apply

18. Low literacy and difficulty explaining complex results (1)
19. Finding participants again (3)
20. Lack of funding (4)
21. Lack of community interest (5)
22. Lack of institutional interest (6)
23. Other (7) ____

Display This Question:

If In your last 3-4 trials, have you targeted trial participants in dissemination of results? = Yes

Or In your last 3-4 trials, have you targeted trial participants in dissemination of results? = In some trials, but not all

Q9 Who was involved in the process?

- o PI (1)
- o Trial coordinator (2)
- o Health workers (3)
- o Translator (4)
- o Other (5) ____

Display This Question:

If In your last 3-4 trials, have you targeted trial participants in dissemination of results? = Yes

Or In your last 3-4 trials, have you targeted trial participants in dissemination of results? = In some trials, but not all

Q10 Do you think the results dissemination worked well for participants?

- o Yes (1)
- o No (2)

Display This Question:

If In your last 3-4 trials, have you targeted trial participants in dissemination of results? = Yes

Or In your last 3-4 trials, have you targeted trial participants in dissemination of results? = In some trials, but not all

And If

Do you think the results dissemination worked well for participants? = No

Q12 If not, why?

____

Display This Question:

If In your last 3-4 trials, have you targeted trial participants in dissemination of results? = Yes

Or In your last 3-4 trials, have you targeted trial participants in dissemination of results? = In some trials, but not all

And Do you think the results dissemination worked well for participants? = Yes

Q16 If yes, why?

____

Q13 Does your institution have formal guidance or an SOP for dissemination of research results?

- o Yes (1)
- o Maybe (2)
- o No (3)

Display This Question:

If Does your institution have formal guidance or an SOP for dissemination of research results? = Yes

Q14 If you would like to share the SOP, please do so here. All identifying and institutional information will be removed

Display This Question:

If Does your institution have formal guidance or an SOP for dissemination of research results? = Yes

Q15 Does the SOP contain specific recommendations or a generic rationale?

- o Generic (1)
- o Specific (2)

Display This Question:

If Does your institution have formal guidance or an SOP for dissemination of research results? = Yes

Q16 Do you find the SOP useful when planning your dissemination?

- o Yes (1)
- o Maybe (2)
- o No (3)

Q17 In your upcoming trials, have you considered dissemination to the participants at the completion of the trial? Please document your plans

____

End of Block: Default Question Block
