# Supplementary material for: Sharing results with participants (and community) in malaria related research: Perspectives and experience from researchers
Source: PLOS Glob Public Health. 2023 Sep 5;3(9):e0002062. doi: 10.1371/journal.pgph.0002062 (PMC10479888; doi:10.1371/journal.pgph.0002062)
Supplement: Attachment — Submitted filename: weston_point by point replies_FINAL.docx [file pgph.0002062.s004.docx]

Dear Editor,

**RE: Review Report for PGPH-D-23-00256: Sharing results with participants (and community) in malaria related research: perspectives and experience from researchers**

**Please kindly find our point by point replies to the reviewer comments below.**

Journal Requirements:

1. Please amend your detailed Financial Disclosure statement. This is published with the article. It must therefore be completed in full sentences and contain the exact wording you wish to be published.

a. State the initials, alongside each funding source, of each author to receive each grant.

*We have added this as suggested.*

b. State what role the funders took in the study. If the funders had no role in your study, please state: “The funders had no role in study design, data collection and analysis, decision to publish, or preparation of the manuscript.”

*We have added the statement as suggested.*

c. If any authors received a salary from any of your funders, please state which authors and which funders.

*We have added this as suggested.*

2. Please provide separate figure files in .tif or .eps format.

*We have reformatted as suggested*

**Reviewer #1**

Thank you for the opportunity to review this interesting manuscript. I find the topic being addressed (return of trial results) quite topical and important. It is also interesting that the authors attempt to place this topic in the broad context of community engagement in research. The paper is fairly well written, although there are a number of minor issues that the authors need to address and or clarify.

1. The manuscripts needs to be proofread to ensure accuracy of language.

***Answer:*** *We have now proof-read the entire manuscript and made relevant changes.*

2. **Introduction**
a) The title of the manuscript suggests that the concern of the paper is about sharing results from trial in Malaria studies. However, immediately after citation number 4, in a spur-of-the-moment, the manuscript seems to digress to ‘community engagement’. The citations that follow focus on broad community engagement. This seeming digression can perhaps be qualified by indicating that the return of trial results is a critical component in CE, then go ahead to speak briefly about community engagement.

***Answer:*** *Indeed we think that dissemination of trial results ultimately is part of a larger community engagement process within research programs. For clarity we have revised the introductory paragraph follows (line 53-65, changes in bold):*

“*While funders, sponsors and researchers are focused on generating best-practice evidence from clinical trials, much less priority is placed on disseminating results to the study participants [3]. Sharing results with participants or involving participants ~~and community members~~ after data collection, analysis and evidence generation overlaps with principles and values borne by participant (community) engagement for ethical global health research [4].* ***Communicating trial results to participant is therefore a critical component of adequate community engagement in clinical research****. Nonetheless, the bulk of clinical trial literature emphasizes the critical role of engagement at the pre- and during- research stages for reasons inherent in facilitating research, improving recruitment, coverage, and adherence [5, 6]. Participant and community engagement to enhance recruitment and adherence alone however ~~is criticized because of its instrumental use that~~ overlooks the broader ethical goals in health research [7]. Engagement at every stage of the clinical trial* ***including at the dissemination stage*** *is considered to facilitate ethical goals such as respecting participants, improving public trust in health research, building community capacities (promoting health and research literacy) and promoting the bi-directional flow of knowledge [4, 5, 8].*

b) In the sentence which begins on p.4 and ends on p.5, there is a phrase, “much less is known about whether and how dissemination of results is done …”. Instead of “whether” it should be ‘the extent to which’. Asking ‘whether’ is so limiting because with this question, you simply need at least only one instance in which the results were shared to get an affirmed answer, and yet I think we should be mainly interested in how often trial results are shared.

***Answer:*** *Although asking ‘whether trial results are shared’ is limiting and binary, it is relevant to understand the baseline. But we agree with the reviewer that it is likely more important to understand the extent of information sharing. We have therefore revised the sentence (line 74-77) as follows:*

*“Although results sharing with participants is widely acknowledged to be an ethical practice in research, much less is known about ~~whether~~****, to what extent*** *and how dissemination of results is done to study participants or the community”*

c) Study aim and objectives: The stated aim in the abstract is somewhat different from that in the rest of the work beginning from the introduction. For example, in the abstract the authors talk of “… methods and strategies” while in the introduction they talk of “… methods or strategies”. Again, “Perspectives” are indicated in the abstract but not in the introduction, while “attitudes” are indicated in the introduction but not in the abstract. (The listing of the results themes on p.6 Par. 1 suggests that the authors’ objectives are around “general opinion” (attitudes?), “Practices” and “Perspectives”).

***Answer:*** *We appreciate the reviewers suggestion to ensure consistency across different parts of the manuscript in relation to the aims. We have now revised the abstract and the last paragraph of the introduction to state the aims as follows:*

- *Identify and document current practice of result dissemination*
- *Explore attitudes towards results dissemination*
- *Understand previous experiences with and barriers to result dissemination*

d) Further, in the introduction the authors have what they call “a secondary aim”. Why are they downplaying this aim and yet there are substantial and significant results for it in the manuscript? In addition, while stating this “secondary aim” the authors talk of “… experiences or barriers …”. Does the use of a disjunction ‘or’ between these words imply that they are synonyms or alternatives? Generally, there is a need to be consistent and clear, including conceptually, about the aims and objectives of the manuscript/study. The main concepts of which the distinctions are not clear in this work are: Attitudes; perceptions, perspectives, and general opinion. For example, is ‘general opinion’ …” conceptually identical with ‘attitude’, or ‘perspective’?

***Answer:*** *Thank you for pointing out the need for distinctions. We have now used the word attitude to be specific for the study objective in abstract and main text, but to have a broader relevance we use a loose term ‘perspectives’ in the title. We believe the objectives specify what within the ‘perspectives’ is the focus of this study.*

3. **Methods**
1) On p. 8, par. 2, while describing webinar attendees, the authors indicate that attendees “*represented* South Asia, Southeast Asia …”. Is the concept of ‘representation’ being used in its strict sense or to mean that the participants simply ‘came from’ these various regions or even one or two countries from each of these regions? If the authors are sure about the representativeness of these attendees, they should provide further information to qualify this claim of representation.

***Answer:*** *We have revised this sentence now for clarity and it reads as follows (line 164):*

*The webinar attendees* ***were*** *from South Asia, Southeast Asia, Sub-Saharan Africa, and Australia.*

2) Still on 8, par. 2, the sample size (11 interviews leading to data saturation) seems to have been determined after data analysis as opposed to the usual norm of determining such saturation during interviews. This casts doubt on the credibility of their claim of data saturation.

***Answer:*** *The study utilized a sequential design with the qualitative component aimed to inform the quantitative data collection. Nonetheless, to reach data saturation, we used a pragmatic approach. First, participants in this study were key informants (a selection of relevant information bearers) as they had experience and involvement in malaria clinical trials. Key informant-participants in this study reassure us of the credibility of our findings. Secondly, the preliminary findings were discussed among webinar attendants that consisted of relevant participants who were involved in, and had experience of, malaria clinical trials. The webinar discussion was utilized as a proxy focus group discussion where we asked if there were any issues, or topics that were missed in our findings. No new topics and themes were found from this webinar other than discussions on their experience that resonated with our findings. Third, we also contacted experts in the field and some of the webinar attendants (see acknowledgements) for informal discussion which also resonated with our findings, and no new topics or themes were identified. Fourth, provisions for further qualitative data collection were outlined in the protocol if saturation was not deemed to be achieved. Fifth, the qualitative data is triangulated with quantitative data that also resonates with the findings. Based on these pragmatic steps, we firmly believe that the qualitative data are thoroughly saturated in this study.*

4. **Results:**
a) On p. 8, par. 3, for the qualitative phase of the study, the investigators/authors mailed (invited) only 13 potential participants even though we know that saturation can sometimes be achieved at 25 interviews, or even more. Why did they target only 13, and what was the approximate number of the target study population?

***Answer:*** *Please kindly see our answer to the comment above.*

b) For the quantitative (survey) phase of this study, the sampling frame was 293 potential participants and ultimately only 44 were able to participate. Unfortunately, the authors do not indicate what the actual target sample size was, how it was determined and hence, whether a sample of 44 sufficiently powered the results.

***Answer:*** *The sampling frame of 293 participants was based on all identified participants. Indeed, a response rate of 44 is low and we have discussed this in the limitation section.*

c) On p. 9, under rationale, par 2, the authors state that “Respondents of the SSIs agreed in principle that results ….”. It is important to use specific quantifiers to give an idea about the extent to which this agreement was shared among the respondents, e.g. most, majority, a few, an almost equal number etc. ‘Some’ can be used but cautiously because apart from indicating ‘at least one or all) it does not give a good picture of how widespread the opinion is.

***Answer:*** *Revised as suggested. Sharing data with participants was considered a good practice by most of the respondents. Unlike in quantitative analysis (percentage or proportion), we used the prominence of the theme here and most resonated their opinion about sharing the data. We have revised the manuscript throughout to add clearer qualifiers.*

d) Some sections of the findings such as on pp. 12 and 13 verbatim quotes from participants are introduced by verbatim quotes of the questions posed by the interviewer, but this is not the case with other quotes. There should consistency in the style of introducing the quotes, preferably, paraphrasing questions into descriptive statements.

***Answer:*** *We have streamlined this across the manuscript.*

e) In the last line on p.13, the words “participants only” seem to be intended as a noun. If that’s true, then it should be one hyphenated work ‘participants-only’.

***Answer:*** *We have corrected this as suggested.*

f) On p. 14, the second paragraph begins as “This was similarly reported…”. What is “this” referring to? Is it a continuation of the previous paragraph? In the same sentence, in reporting the results in the form proportions or fractions, the denominator is 35, but elsewhere it is 42. Elsewhere, the authors indicate 14/33; 3/33 on p.17, 13/25 on p. 18, 9/40 on p.19, etc. Why? Is the denominator representing the number of people who responded to particular questions or something else? Clarity is needed.

***Answer:*** *Indeed ‘this’ refers to the previous paragraph. We have now changed this so that it is one paragraph only. Indeed the denominator refers to the participants who answered the specific question.*

g) Table 2, pp 15 to 16: What is the rationale of presenting this information in a table form? Secondly, the authors indicate that this (text in the table) is an “author-edited narrative”. This gives an impression that these were paraphrased views of participants. If they are not paraphrased, then they should be written like any other verbatim quotes and the edits they mention can be indicated with appropriate punctuation such as square brackets to indicate that some text was added or skipped, and *sic* where applicable.

***Answer:*** *The authors felt that presenting an illustrative example of dissemination practices is helpful for the reader and easier to read in the table than within the main text. Having it in the table also highlights the uniqueness of this example, while the text tries to reflect the heterogeneity across different sites and investigators. Indeed, the text is an author-edited narrative that summarized the recount of the participant across different section of the interview.*

h) On p. 17, in the middle of the second paragraph, there is a phrase: ..”…time lag was mentioned by the majority (14/33, 42.2%) … These figures are not a majority.

***Answer:*** *We have changes and use the term ‘large proportion’ now (line 365).*

i) On p. 18, the claim in the sentence beginning the paragraph immediately the first direct quote is factually contentious. It is not true, as far I know, that rural populations in Africa are generally more mobile than those in urban settings. Instead the reverse is generally true. The authors seem to be having mind certain specific populations (exceptions) that fit these attributes (rural and highly mobile), but it would be wrong to generalize at a continental or regional (Sub-Saharan Africa) level. They need to qualify their claim or specify that these were the attributes of the specific populations where the Malaria trials they refer to took place.

***Answer:*** *The quote is actually from a respondent from the Americas. In the text before the quote we state that ‘malaria trials are often conducted in remote locations where populations can be highly mobile.’ We feel this is sufficiently specific to malaria trials independent of location to ensure this statement is not generalized.*

5. **Discussion**
a) P. 20, second sentence (third line) “… deemed to have rights to know …”. The concept of rights can be controversial. It can be replaced with ‘deserve to know’ except if the concept of ‘rights’ was explicitly introduced by the respondents. If this statement on rights is intended to be a normative one as opposed to descriptive, it is fine but it needs to be clear.

***Answer:*** *‘Rights’ were explicitly used by our respondents. We agree that the term ‘rights’ is controversial, but ethicists do seem to support that participants have ‘rights’ to know the outcome of the research they participated unless the sharing of results can do harm to them (Markman M. Providing research participants with findings from completed cancer‐related clinical trials: Not quite as simple as it sounds. Cancer. 2006 Apr 1;106(7):1421-4 and Beauchamp TL, Childress JF. Principles of biomedical ethics. Edicoes Loyola; 1994.)*

*At the same time fulfilling respect for participants obligates the researcher to provide a summary of research results on completion of the study (Fernandez CV, Kodish E, Weijer C. Informing study participants of research results: an ethical imperative. IRB: Ethics & Human Research. 2003 May 1;25(3):12-9.)*

b) P. 20, second par, the first statement is clearly reporting the findings of this study but at its end there is a citation! This is common throughout the discussion section. What does this mean?

***Answer:*** *Citations are used throughout the discussion to put our findings into the larger perspective of the available literature. We have revised throughout to clearly distinguish what our findings are and what reflection refers to the overall body of literature.*

c) P.21, par 2, last sentence: I find the concept of ‘epistemic trust’ unfamiliar. Generally there is a growing tendency to misuse use the concept of ‘epistemology’ (such as the growing rhetoric about ‘epistemic justice/injustice’). Since it is clear that what the authors are referring to is the increasing ‘doubt about the credibility of research results’ they can choose to replace the phrase ‘declining epistemic trust’ with a different phrase such as ‘increasing allegations of lack of scientific integrity, or credibility of research data/findings’ to avoid potential conceptual controversies.

***Answer:*** *We have revised the sentence in line with the suggestion. It now reads as follows (line 480-482):.*

*‘Declining ~~epistemic~~ trust* ***in scientific integrity*** *and emergence of science denialism globally further urges the scientific community to invest into overall community/participant engagement’*

d) P. 23, last par. The paragraph is introduced as limitations of the study, although it is not clear how the content of this particular paragraph can be regarded as limitations.

***Answer:*** *We have removed the introductory sentence and replaced it with the following sentence (line 539): This study attempted to utilize the formative approach to build evidence incorporating a mixed methods approach.*

e) Generally, the discussion could have been better. A lot of space is dedicated to paraphrasing the results of the study and showing how they are corroborated by results from other similar studies. Whereas this is part of what needs to be done in the discussion section, there is need to directly draw inferences and or implications from the results – something like what has been done on p. 24, second paragraph. In this paragraph, the authors have done quite well in attempting to draw the implication of very low enthusiasm on the part of researchers to respond to their request to participate in the study – that is, that it could be that researchers do not find the topic of sharing trial results to be of priority. (Disclaimer about this comment: This type (not necessarily quality) of discussion is common in many empirical Social Science papers, and yet my background is philosophy where there is a lot of emphasis on independent examination of the reported views, and drawing implications and or further inferences from them).

***Answer:*** *Thank you for your feedback. We agree with your overall feedback and also believe that it may be the inter-disciplinary differences that may have influenced the content. Nonetheless, we have revised the discussion where relevant.*

**Reviewer #2**

This study examines an important yet complex area of participant engagement and highlights a departure from optimal research practices.

1. Data availability: The requirement for data availability is now standard and expected so this requirement should be considered at the research design stage. Have the researchers considered removing identifying information from the qualitative data so that the data may become available to other researchers? This is important for both research transparency and re-use of data. The above suggestion should be considered in conjunction with the following observation. The researchers presumably assured participants that data collected would be secure and that confidentiality would be maintained. However, the use of Otter.ai does not seem entirely compatible with these assurances on a close reading of the Otter.ai privacy policy (See <https://otter.ai/privacy-policy>).

***Answer:*** *We have indeed considered making the data available, but even when identifying information about the respondent is removed, it is relatively easy for people working in the area of malaria clinical trials to identify respondents based on the examples of trials and locations they provide. We anticipated this at the design stage and therefore didn’t want to make the data open access. During the informed consent process, consent was obtained to record and transcribe the interviews verbatim. While Otter.ai has access to the researchers name and identifying information, the interviews that were uploaded for transcription do not contain the full names nor details of the respondents.*

2. It would be useful to explicitly state what kind of results the researchers are discussing. Are these individual-level results or overall research findings, or both? The distinction is made by some research participants but a clarification from the start of the manuscript would seem appropriate.

***Answer:*** *Indeed we aimed at exploring dissemination for overall study results and have clarified this further now at the end of the introduction when we talk about the aims of the study. However it is correct that a small number of the research participants conflated the two and we discuss this in the discussion section.*

3. Page 10: The statement in the findings ‘Few respondents were doubtful of the rationale of sharing results with the community members, particularly questioning how sharing of results would yield tangible impacts such as policy change or incorporation of alternative treatment regimens in their community.’ should firstly be clarified and then commented on in the Discussion.

a. The clarification required is whether the researchers intended ‘Few’ = not many or ‘Some’= a few.

***Answer:*** *We have clarified now and changed to ‘some respondents’*

b. Following on from the above, ‘Few respondents were doubtful of the rationale’ means ‘Not many of the respondents were doubtful of the rationale…’. In other words, many respondents understood and supported the rationale. I would suggest it be changed to ‘Some respondents questioned the rationale for sharing results with community members and specifically expressed doubts about how sharing results would yield tangible impacts, such as policy change or incorporation of alternative treatment regimens in their community.’

***Answer:*** *We have followed the advise and changed as suggested.*

c. The reason this statement should be commented on in the Discussion is because the research participants who voiced this view have conflated the ethical requirement to share findings in order to show respect for participants, build trust etc. with the social benefits all research is required to promote. This, in itself, is an important finding (even if only very few voiced the view).

***Answer:*** *Thank you for the suggestion. We agree with you and we have added a line in discussion (line 451-455).*

*“Although disseminating results may not offer tangible impacts such as policy change or provision of alternative treatment to the community members, sharing research outcomes serve to address ethical research principles of showing respect to the participants, ensuring transparency and building trust. “*

4. In the Discussion, it would be useful to examine existing literature in conjunction with the findings of this study. That is, in which ways does this study align with the literature and in which ways does the data in this study illuminate areas previously not explored/understood etc.? Leaving this task to the reader is not ideal.

***Answer:*** *Thank you for the feedback. We explored the literature to look for similar studies, for instance third paragraph describes a large survey conducted among clinical trial investigators that demonstrates a significantly low level of attitude towards results dissemination and even lesser in their practice. That also demonstrates there is a paucity in this particular aspect of research engagement. Our study findings have implications that have broader and deeper impacts which we have discussed in subsequent paragraphs, for e.g. what are barriers/challenges for researchers in disseminating the trial results. Subsequently, we discuss the potential solutions that could be forged from the findings of this study.*

6. Page 23: The paragraph following the statement: ‘This work has several limitations.’ is certainly not a limitation of any kind and should not be stated as such. This section relates to the researchers’ reflecting on the qualitative research (perhaps because they had originally undervalued the contribution that qualitative data makes?). The statement about limitations belongs in the next paragraph relating to the low response rate for the survey.

***Answer:*** *We agree with the reviewer. We have revised and added a statement to reflect how we utilized the formative approach to build the evidence incorporating a mixed methods (line 539-540).*

7. COREQ Checklist: Could the researchers please double-check the list? The question ‘How many people refused to participate or dropped out? Reasons?’ does not appear to have been completed.

***Answer:*** *Thank you. COREQ has been revised.*

Minor points

8. There are multiple minor errors/issues, which should be corrected so as not to distract the reader. A closer careful reading of the text would be helpful
1. Would suggest 'results-sharing' to avoid confusion.

***Answer:*** *We have changed this throughout the manuscript.*

2. Page 6: should be ‘sharing results with’- pleach check multiple instances.

***Answer:*** *We have changed this throughout the manuscript*

3. Page 6: New sentence at ‘for example, a few minutes were invested in building rapport before commencing each interview.’

***Answer:*** *We have changed as suggested.*

4. Page 10: ‘Echoing with the qualitative interviews…’ ‘with’ should be removed.

***Answer:*** *We have changed as suggested.*

5. Page 11: ‘reported to have disseminated…” should be ‘reported disseminating…’

***Answer:*** *We have changed as suggested.*

6. Page 14: the following needs to be amended ‘reporting about participants only meetings’

***Answer:*** *We have changed to participant-only meetings for clarification*.

7. Page 14: Perhaps clarify what was intended with ‘print outs’. Were these printouts disseminated to community members?

***Answer:*** *We have changed as suggested.*

8. Page 14: ‘sharing findings in an individual level’ should be ‘sharing findings at an individual level’.

***Answer:*** *We have changed as suggested*

9. Page 21: New sentence required at: ‘for example sharing individual results due to issues of confidentiality in HIV research and challenges…’

***Answer:*** *We have changed as suggested*

10. Page 21: ‘invest into’ needs to be changed to ‘invest in’

***Answer:*** *We have changed as suggested*

11. Pages 21-22: the following statement is not very clear ‘Unfortunately, such post-trial commitments and engagement with participants is underwhelmingly executed and has demonstrated impacts on current research practice.

***Answer:*** *We have revised the sentence for clarify (line 493) it now reads as follows*

“*Unfortunately, such post-trial commitments and engagement with participants is underwhelmingly executed and has ~~demonstrated~~* ***negative*** *impacts on current research practice. “*
