# Supplementary material for: Sharing results with participants (and community) in malaria related research: Perspectives and experience from researchers
Source: PLOS Glob Public Health. 2023 Sep 5;3(9):e0002062. doi: 10.1371/journal.pgph.0002062 (PMC10479888; doi:10.1371/journal.pgph.0002062)
Supplement: Attachment — Submitted filename: Weston_point_by_Point2.docx [file pgph.0002062.s005.docx]

Dear Editor,

RE: Review Report for PGPH-D-23-00256: Sharing results with participants (and community) in malaria related research: perspectives and experience from researchers

Please kindly find our point-by-point replies to the remaining minor reviewer comments below.

**Editors comments**

Please make the minor revisions suggested by R1. In addition, please revisit the comments of R2 on the previous version of this manuscript, especially his comments related to Discussion section as mentioned by the reviewer on this version.

***Answer:*** *as discussed via email there are only comments from reviewer #2 available and we outline below how we have addressed them.*

**Reviewer #2:**

The paper is much improved even though the researchers did not seem to fully understand the suggestion made by Reviewer 2 regarding the Discussion.

The researchers should consider including a heading at line 547. For example, it could read 'Researchers’ Reflections'. In addition, a heading, such as 'Conclusion', should be added at line 570. The addition of these headings would facilitate reading the manuscript.

***Answer:*** *We have now added subheadings researchers’ reflections section and conclusion as requested. In terms of reflexivity of the researchers, we have followed COREQ guidelines to state the academic background of the authors (please see heading ‘data collection’ in the Methods section), that informs the positionality of the authors in relation to the topic. Nonetheless, to add more clarity under the heading of ‘Researchers’ reflections’ we have explained how researchers’ position, and background may have influenced the interpretation presented in the study. This now reads as follows (line 532-537)****:***

“*Inherent in interpretive methods utilized in this study, the findings and their interpretations are also influenced by the personal and professional values including experiential knowledge of the authors. In future, interpretive methodology could be broadened to include the opinions of community and stakeholders related to the findings presented in this study. In addition, to respond to the low survey participation incentivized online surveys could be a possible way to enhance the number and diversity of participants.”*
